# β-Hexachlorocyclohexane Drives Carcinogenesis in the Human Normal Bronchial Epithelium Cell Line BEAS-2B

**DOI:** 10.3390/ijms22115834

**Published:** 2021-05-29

**Authors:** Elisabetta Rubini, Marco Minacori, Giuliano Paglia, Fabio Altieri, Silvia Chichiarelli, Donatella Romaniello, Margherita Eufemi

**Affiliations:** 1 Department of Biochemical Sciences “A.Rossi-Fanelli”, Sapienza University, P.le A.Moro 5, 00185 Rome, Italy; elisabetta.rubini@uniroma1.it (E.R.); marco.minacori@uniroma1.it (M.M.); giuliano.paglia@uniroma1.it (G.P.); fabio.altieri@uniroma1.it (F.A.); silvia.chichiarelli@uniroma1.it (S.C.); 2 Enrico ed Enrica Sovena Foundation, 00199 Rome, Italy; 3 Department of Experimental, Diagnostic and Specialty Medicine (DIMES), “Alma Mater Studiorum”, University of Bologna, 40138 Bologna, Italy; donatella.romaniello@unibo.it

**Keywords:** β hexachlorocyclohexane (β-HCH), carcinogenesis, organochlorine pollutants (OCPs), signal transduction

## Abstract

Organochlorine pesticides constitute the majority of the total environmental pollutants, and a wide range of compounds have been found to be carcinogenic to humans. Among all, growing interest has been focused on β-hexachlorocyclohexane (β-HCH), virtually the most hazardous and, at the same time, the most poorly investigated member of the hexachlorocyclohexane family. Considering the multifaceted biochemical activities of β-HCH, already established in our previous studies, the aim of this work is to assess whether β-HCH could also trigger cellular malignant transformation toward cancer development. For this purpose, experiments were performed on the human normal bronchial epithelium cell line BEAS-2B exposed to 10 µM β-HCH. The obtained results strongly support the carcinogenic potential of β-HCH, which is achieved through both non-genotoxic (activation of oncogenic signaling pathways and proliferative activity) and indirect genotoxic (ROS production and DNA damage) mechanisms that significantly affect cellular macroscopic characteristics and functions such as cell morphology, cell cycle profile, and apoptosis. Taking all these elements into account, the presented study provides important elements to further characterize β-HCH, which appears to be a full-fledged carcinogenic agent.

## 1. Introduction

Ecological balance is the result of a mutual relationship between living creatures and their ecosystem: each organism, in fact, is capable of rapidly responding and adapting to external perturbations, maintaining a dynamic equilibrium with the surroundings. Therefore, a healthy environment represents the sine qua non condition to ensure the physiological homeostasis of biological systems, especially for human beings.

The environmental presence of pesticides, industrial waste, exhaust gas, particulate matter, and other xenobiotic substances has been universally considered to be accountable for many pathological conditions, including cancer [1,2,3,4]. The correlation between synthetic chemicals and carcinogenesis was originally identified in the middle 1700s [5], but it was only in 1915 that Yamagiwa published the first experimental study on cancer pathogenesis in association with environmental contaminant exposure [6]. Outcomes from this research confirmed Shimkin’s hypothesis and consolidated the observations made in some epidemiological investigations, dating from the 18th and 19th centuries, which established the occurrence of snuff-related malignant lesions of nasal mucosa in smokers [7] and the development of bladder cancer in workers employed in chemistry and rubber industries [8]. Moreover, the International Agency for Research on Cancer (IARC) has so far classified 120 agents as carcinogenic to humans [9]. Several scientific papers published in the last few years demonstrated that chemical substances do not necessarily exhibit oncogenic properties taken individually, but they may reveal dangerous effects in combination with other compounds owing to additive and synergistic effects, even at low doses [10]. 

Another aspect that should not be neglected is the potential bioaccumulation under which some persistent compounds can progressively reach high and unpredictable concentrations inside organisms, leading to significant systemic toxicity [11]. 

Organochlorine pesticides (OCPs) constitute the majority of the total environmental pollutants and include among their members TCDD, HCH, PCBs, bisphenol A, and dieldrin [12]. The carcinogenicity of the most popular OCPs has already been established, but significant gaps still remain in the knowledge of less common compounds [13,14]. During the last decades, growing interest has been focused on β-hexachlorocyclohexane (β-HCH), a secondary product from the industrial synthesis of the best-known lindane (γ-HCH), which is the only HCH isomer with insecticidal properties. Lindane was extensively used as a pesticide for agricultural purposes until the late 1960s, but was then banned in several countries and blacklisted in 2009 by the Stockholm Convention [15,16]. Unfortunately, the environmental persistence of hexachlorocyclohexane isomers represents a current global problem because of their illegal disposal in uncontrolled dumps all around the world [17]. In particular, β-HCH is virtually the most hazardous member of the HCH family due to its physicochemical characteristics: its impressive stability, reflected in the considerable residual amount remaining in the environment, enables β-HCH to be defined as the “fossil” of technical-grade lindane [18]. For this reason, beside developing and investigating efficient remediation strategies, it appears to be absolutely essential to deepen the knowledge of β-HCH’s biological impact. For the past five years, our research group has been focused on the molecular mechanisms underlying β-HCH toxicity. Cellular studies carried out on a panel of cancer cell lines contributed to elucidating β-HCH’s activation pathways, identifying the protein STAT3 (Signal Transducer and Activator of Transcription 3) as the main modulator in the molecular responses induced by this organochlorine [19]. Further investigations shed some light on the potential activity of β-HCH as an endocrine-disrupting chemical, assessing its capability to activate AhR signaling and to promote other cellular functions related to redox homeostasis and energy metabolism [20]. 

Taking all of these elements into account, it is questionable whether β-HCH could also trigger cellular malignant transformation toward cancer development. For this purpose, human continuous cell line BEAS-2B (normal bronchial epithelium) was tested with 10 µM β-HCH. In accordance with our previous studies, the experimental concentration of the pesticide was chosen by averaging across all the plasma concentration values detected in patients under a biomonitoring study carried out in the Valle del Sacco [21], in order to evaluate both the genotoxic and non-genotoxic effects of β-HCH on cancer initiation by reproducing real exposure conditions.

## 2. Results

The non-genotoxic and indirect genotoxic effects of organochlorine pesticides are expressed through various intracellular events, ranging from hyperproliferation to the activation of oncogenic signaling pathways responsible for growth factor release and reactive oxygen species (ROS) production [22].

ROS have been reported to cause different forms of DNA damage by several mechanisms, either as mediators or direct inducers [23]. For this reason, ROS may be accountable for the indirect genotoxic action of β-HCH, which triggers biochemical responses that significantly affect cellular macroscopic characteristics and functions such as cell morphology, cell cycle profile, and apoptosis.

### 2.1. β-HCH Increases Cell Proliferation

**Figure 1:**
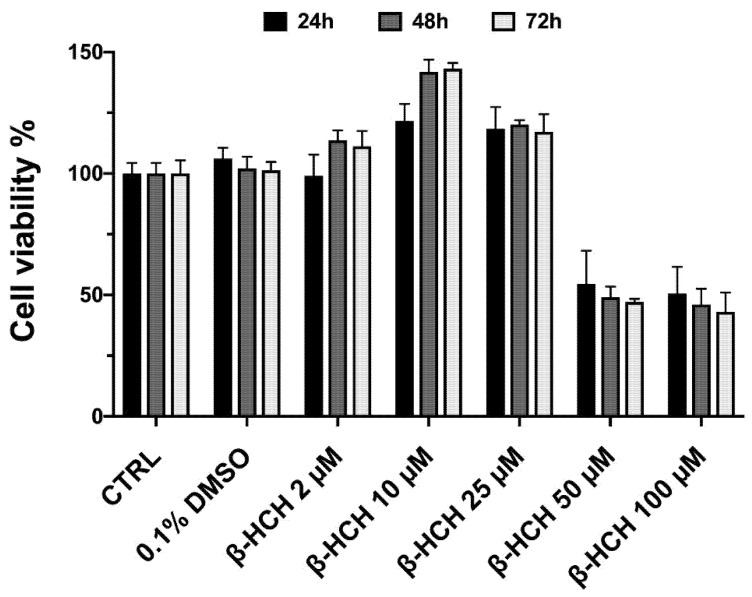
MTT assay on BEAS-2B cells exposed for 24, 48, or 72 h to β-HCH within a concentration range from 2 µM up to 100 µM.

The level of toxicity of a substance and its potential impact on cell proliferation can be assessed through the MTT assay. In order to evaluate the cytotoxic effect of β-HCH, BEAS-2B cells were incubated with β-HCH for 24, 48, and 72 h within a range of concentrations from 2 µM to 100 µM; it is worth emphasizing that the value of 10 µM, used as the experimental concentration, corresponds to the plasmatic levels of β-HCH detected in patients taking part in an epidemiological study in the Valle del Sacco [21]. The histogram reported in the figure clearly shows that a cytotoxic effect occurred only at 50 and 100 µM, whereas 10 µM β-HCH seemed to induce proliferative activity. In fact, cell viability increased by 20% at 24 h following 10 µM β-HCH treatment, and this trend was even more noticeable after 48 h, where the percentage of vital cells was 40% higher compared to the untreated sample taken as a control. Similar outcomes were observable after 72 h of incubation (Figure 1).

### 2.2. β-HCH Can Activate Oncogenic Signaling Pathways

**Figure 2:**
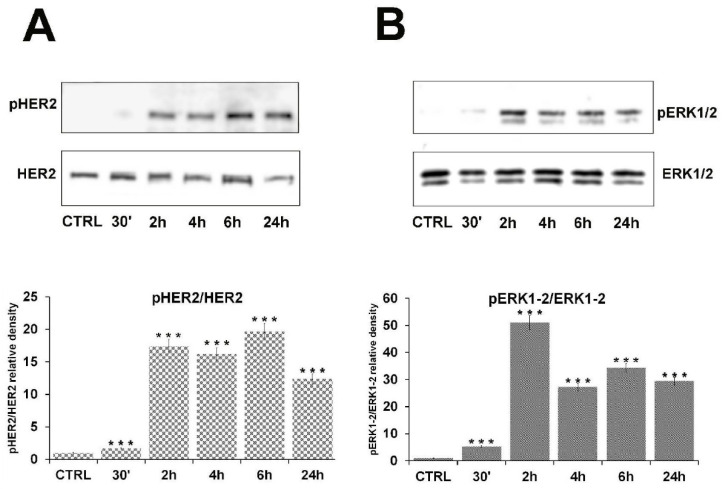
Time course assay on BEAS-2B cells. Cells were treated with 10 µM β-HCH for a period from 30 min up to 24 h. Extracts were processed by immunoblotting and incubated with the indicated antibodies. The phosphorylation of HER2 (**A**) and ERK1/2 (**B**) occurred after 30 min of incubation, as clearly demonstrated by Western blot. HER2 and ERK1/2 phosphorylation levels were measured on the amount of total HER2 and ERK1/2, respectively, present in each sample and were referred to the control. Experiments were repeated three times with similar results, and the obtained values are presented as the mean and standard deviation. Statistical analysis was performed with GraphPad Prisma software using Student’s *t*-test. Statistically significant differences (*** *p* < 0.001) are marked with asterisks and are referred to the control.

The proliferative response of cells to 10 µM β-HCH strongly suggests the activation of oncogenic signaling pathways. For this reason, a time course assay was performed on BEAS-2B cells treated with 10 µM β-HCH for increasing incubation time, from 30 min to 24 h. Total protein extracts were analyzed by Western blot by using specific antibodies against proteins involved in growth factor signaling cascades. The obtained results (Figure 2) reveal the activation of HER2 (Figure 2A) and ERK1/2 (Figure 2B).

### 2.3. β-HCH Induces EGF Release

The observed increase in proliferation and the activation of oncogenic transduction pathways led to the hypothesis that β-HCH might induce an autocrine signaling that enables cells to proliferate even in the absence of external stimuli due to an improper synthesis of growth factors. This assumption was confirmed by investigating the possible β-HCH-dependent release of Epidermal Growth Factor (EGF). After treating BEAS-2B cells for 24–48 h with 10 µM β-HCH, an aliquot of conditioned medium was subjected to dot blot using a specific antibody against EGF.

**Figure 3:**
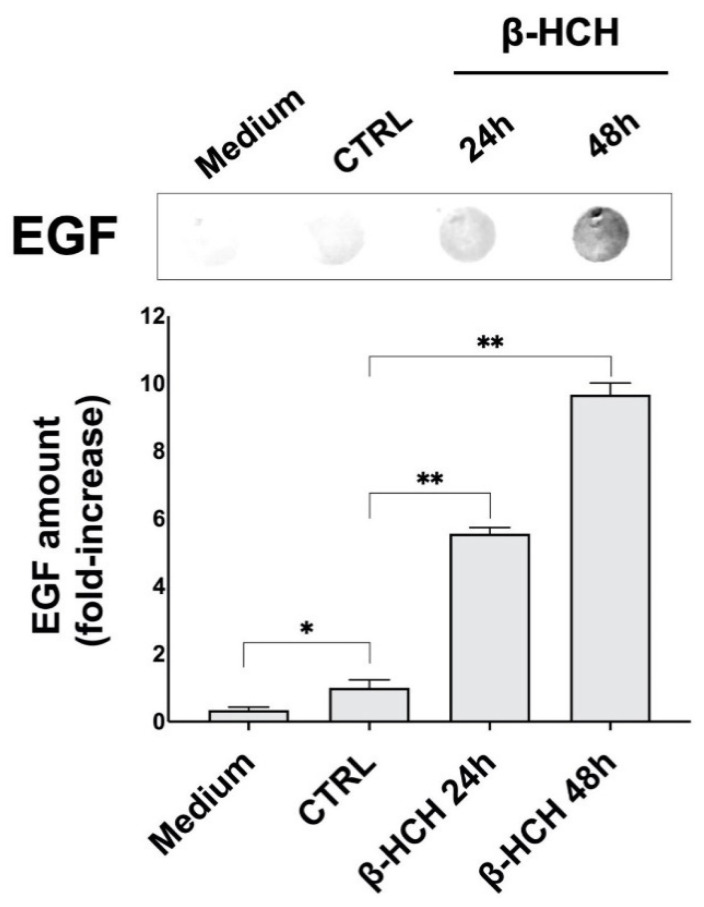
Dot blot analysis for EGF secretion in BEAS-2B conditioned medium after a 24–48 h treatment with 10 µM β-HCH. An aliquot of 500 µL of medium from stimulated BEAS-2B cells (1.5 × 10^5^ cells/mL) was loaded on a nitrocellulose membrane, and EGF was detected using a specific primary antibody through immunoblotting. The obtained signal was subjected to densitometric analysis and the EGF fold-increase was estimated compared to the control. Experiments were repeated three times with similar results, and the obtained values are presented as the mean and standard deviation. Statistical analysis was performed with GraphPad Prisma software using Student’s *t*-test. Statistically significant differences (* *p* < 0.05; ** *p* < 0.005) are marked with asterisks and are referred to the control.

Figure 3 displays the dot blot carried out on BEAS-2B conditioned medium to detect EGF presence. The experimental outcomes demonstrate that β-HCH induced the release of EGF in the culture medium of cells after 24–48 h; the increase in EGF production was assessed by densitometric analysis of the EGF signal.

### 2.4. β-HCH Boosts ROS Production

The overproduction of ROS is a distinctive feature of cancer cells and is critically involved in the mechanisms responsible for malignant transformation. ROS, in fact, can directly or indirectly contribute to cancer initiation and progression, leading to the activation of oncogenic transduction pathways (i.e., HIF-1α, NF-kB) or causing DNA damage [24]. Therefore, as part of the investigation on the potential carcinogenicity of β-HCH, variations in ROS levels were evaluated as a possible consequence of the increased proliferation rate and EGF release.

**Figure 4:**
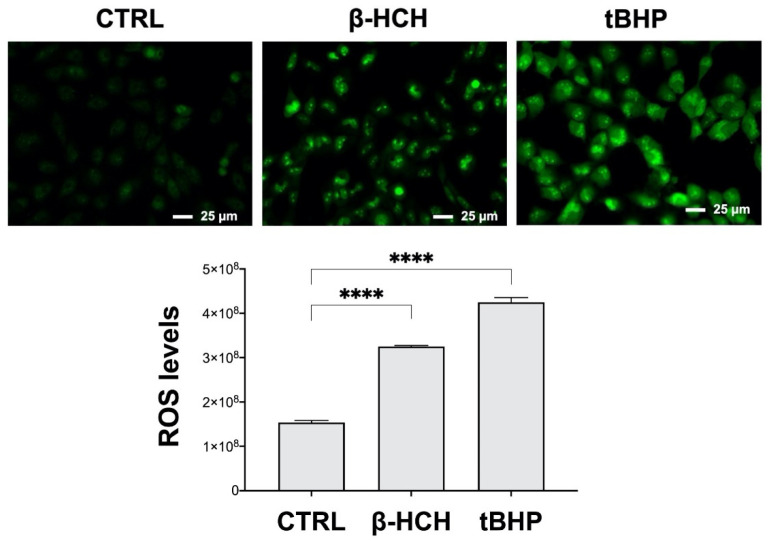
β-HCH induces ROS production. The images and the relative fluorescence quantification reported in the histogram clearly show an increase in ROS levels upon treatment with 10 µM β-HCH for 48 h compared to the control; 75 µM tBHP for 1 h was used as a positive control. Fluorescence intensity was then quantified by averaging across the CTCF (Corrected Total Cell Fluorescence) calculated with ImageJ. Images were captured under the same acquisition parameters and are representative of three independent experiments. Results are presented as the mean and standard deviation. Statistical analysis was performed with GraphPad Prisma software using Student’s *t*-test. Statistically significant differences (**** *p* < 0.0001) are marked with asterisks and are referred to the control.

ROS detection was determined by immunofluorescence using the CellROX reagent. Cells stimulated with tert-butyl hydroperoxide (tBHP) were taken as a positive control. As displayed in Figure 4, an increase in fluorescence intensity occurred upon both β-HCH and tBHP treatments (details in the figure legend), attesting to the production of ROS; this result is further highlighted by the histogram.

### 2.5. Genotoxic Responses to β-HCH

As already documented in the scientific literature, the major target of ROS is represented by DNA electron-rich bases, which undergo oxidation, generating a wide range of genotoxic modifications [25].

To verify whether the oxidative stress conditions induced by β-HCH could promote cellular genotoxic responses, the status of suitable markers was examined. In particular, cells were tested for γH2A.X, a DNA double-strand break sensor [26]. Total protein extracts from BEAS-2B cells, treated with β-HCH for different incubation times, were subjected to immunoblotting using specific antibodies against γH2A.X; tert-butyl hydroperoxide was employed as a positive control.

**Figure 5:**
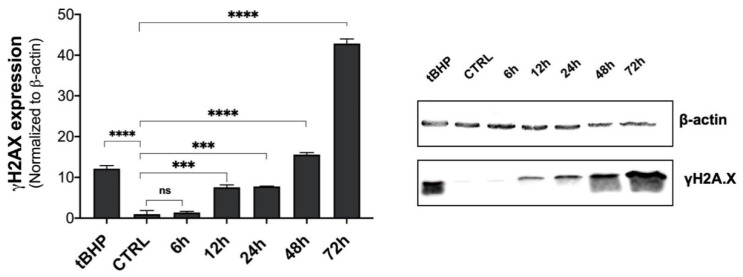
β-HCH induces the phosphorylation of H2A.X on Serine 139 in BEAS-2B cells. Total protein extracts were obtained from cells exposed to 10 µM β-HCH at different time points. Treatment with 75 µM tBHP for 1 h was used as a positive control for DNA damage. A specific primary antibody was used to detect pS^139^H2A.X, and β-actin was assessed as a loading control protein. Experiments were repeated three times with similar results, and the obtained values are presented as the mean and standard deviation. Statistical analysis was performed with GraphPad Prisma software using Student’s *t*-test. Statistically significant differences (*** *p* < 0.001; **** *p* < 0.0001) are marked with asterisks and are all referred to the control.

The blot result, together with the relative densitometric analysis (Figure 5), confirmed H2A.X phosphorylation in response to 10 µM of β-HCH, attesting to the occurrence of genotoxic events temporally coincident with ROS production.

**Figure 6:**
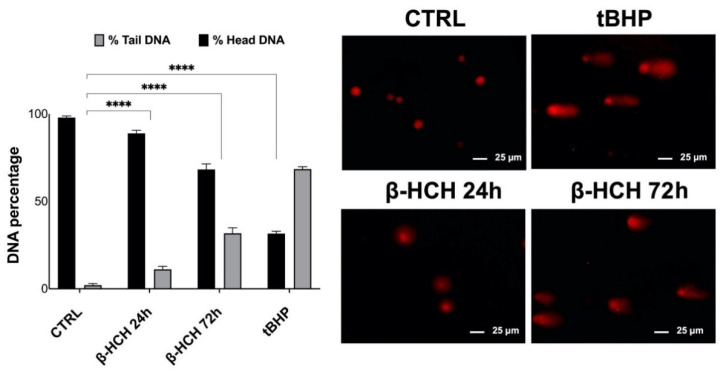
DNA fragmentation increases upon β-HCH treatment. Comet assay was performed on BEAS-2B cells exposed for 24–72 h to 10 µM of β-HCH; 75 µM of tBHP for 1 h was employed as a positive control. The extent of DNA fragmentation was assessed by quantifying the percentage of DNA in the comet tail using CaspLab software. As shown in the figure and confirmed by the histogram, the percentage of tail DNA rose after 72 h of incubation with β-HCH, testifying to the occurrence of a genotoxic response. Experiments were repeated three times with similar results, and the obtained values are presented as the mean and standard deviation. Statistical analysis was performed with GraphPad Prisma software using Student’s *t*-test. Statistically significant differences (**** *p* < 0.0001) are marked with asterisks and are all referred to the control.

To confirm that the phosphorylation of H2A.X correlates to effective DNA damage, comet assay [27] was performed on BEAS-2B cells exposed for 24 and 72 h to 10 µM β-HCH; tBHP was used as a positive control for the experiment. As evident in the figure, β-HCH caused a rise in the percentage of DNA in the comet tail, especially after 72 h, testifying to the fragmentation of DNA consistent with ROS-induced damage (Figure 6).

### 2.6. β-HCH’s Impact on Cell Cycle and Apoptosis

**Figure 7:**
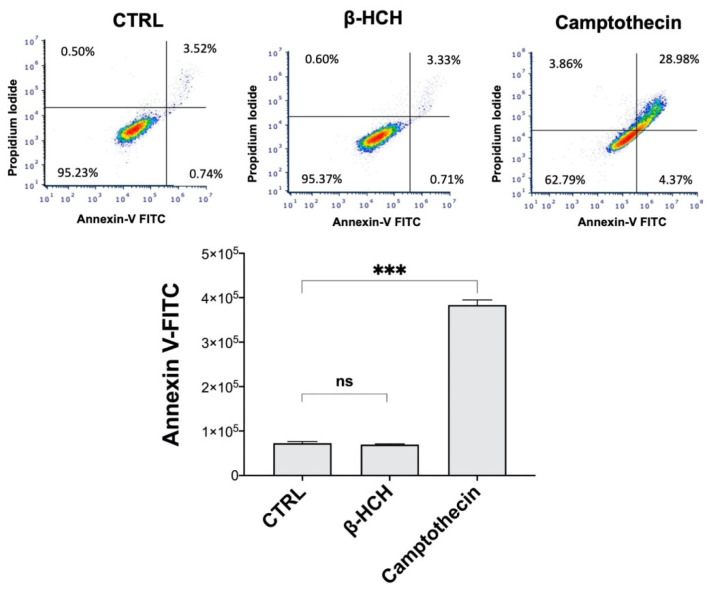
Annexin V-FITC assay performed on BEAS-2B cells treated with β-HCH. Cells were exposed for 48 h to 10 µM β-HCH or 10 µM camptothecin (positive control for apoptosis induction). The results show that β-HCH did not induce apoptosis. Experiments were repeated three times with similar results, and the obtained values are presented as the mean and standard deviation. Statistical analysis was performed with GraphPad Prisma software using Student’s *t*-test. Statistically significant differences (*** *p* < 0.001) are marked with asterisks.

The results obtained so far hint that β-HCH could induce apoptosis inhibition in BEAS-2B. An analysis of the cell distribution by flow cytometry revealed that β-HCH did not affect the apoptotic population (3%), which was instead greater by almost 10-fold in samples treated with camptothecin, the positive control (Figure 7).

**Figure 8:**
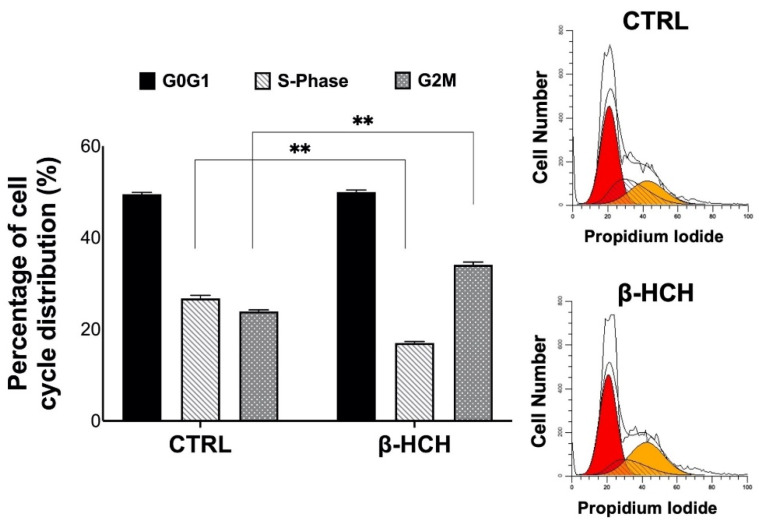
Effects of β-HCH on the cell cycle profile in BEAS-2B cells. Exposure to 10 µM β-HCH for 48 h induced an increase in the percentage of propidium-iodide-stained cells in the G2M phase. This result could relate with the establishment of repair mechanisms that prevent cells from entering mitosis. Experiments were repeated three times with similar results and analyzed using ModFit LT software. Obtained values are presented as the mean and standard deviation. Statistical analysis was performed with GraphPad Prisma software using Student’s *t*-test. Statistically significant differences (** *p* < 0.005) are marked with asterisks.

Moreover, the cell cycle profile obtained after β-HCH treatment displayed a slight accumulation in the G2M phase, which is typical of hyperproliferating cells and sustained ROS production. In this altered context, the G2M checkpoint is induced to enable the intervention of repair mechanisms for damaged neosynthesized or unduplicated DNA (Figure 8).

### 2.7. β-HCH Affects Cellular Morphology

In addition to the presented experimental data, an assessment of β-HCH’s capability to modulate cellular structural plasticity could provide a further element to confirm its carcinogenic potential. Cytoskeletal organization can, in fact, adapt in response to oncogenic stimuli, determining a change in cellular morphology that is aimed at fostering proliferation activity and invasiveness [28].

**Figure 9:**
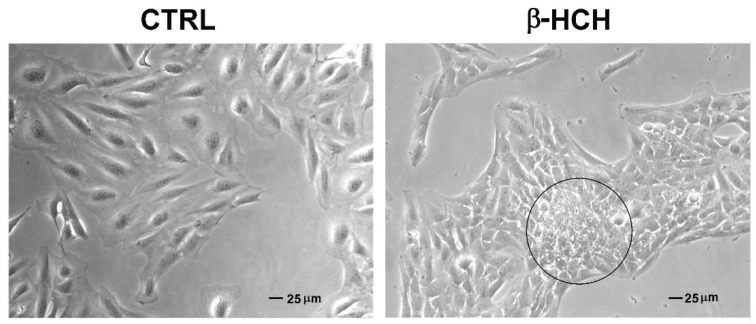
β-HCH-induced morphological changes in BEAS-2B cells. Cells were seeded at 3 × 10^5^ cells/well and, after 24 h, were exposed to 10 µM β-HCH for 48 h. The different cell arrangement induced by β-HCH is evidenced in the black circle. The selected images are representative of three independent experiments.

By looking at BEAS-2B under a microscope, it is possible to notice that cells exposed to 10 µM β-HCH for 48 h grow differently on the well surface, appearing more clustered compared to the untreated sample (Figure 9).

**Figure 10:**
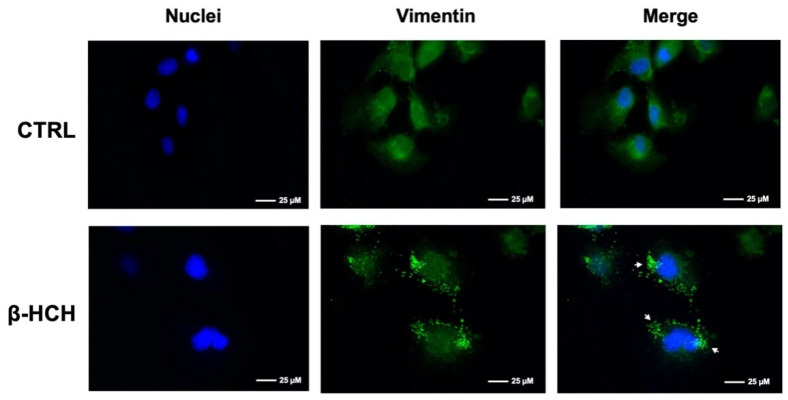
β-HCH induces a rearrangement in vimentin organization. Cells were exposed to 10 µM β-HCH for 48 h and subjected to immunofluorescence analysis using a specific antibody against vimentin. White arrows indicate the punctuated pattern assumed by vimentin in treated cells, testifying to a morphological change. The selected images are representative of three independent experiments and were captured under the same acquisition parameters.

Therefore, cells were immunoassayed for the structural protein vimentin, an indicator of the cytoskeletal health state: vimentin is a well-established marker of epithelial–mesenchymal transition (EMT) and is crucially implicated in the modulation of cytoskeleton dynamics under stress conditions [29]. In BEAS-2B cells stimulated for 48 h with β-HCH and subjected to immunofluorescence, vimentin exhibited a punctuated polymerization pattern (a symptom of aggresome formation) [30], rather than the filament network recognizable in the control (Figure 10). This outcome testifies to protein reorganization toward a morphological change.

### 2.8. β-HCH Modulates the Expression of the Proliferative Marker Ki67

**Figure 11:**
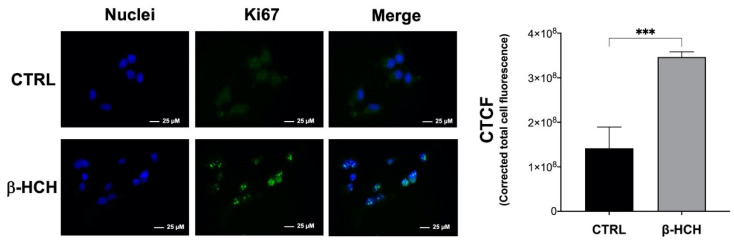
Impact of β-HCH on Ki67 expression in BEAS-2B cells. After a 48 h treatment with 10 µM β-HCH, cells were fixed, permeabilized, and subjected to immunofluorescence analysis using a specific antibody against Ki67. The images clearly show an increase in Ki67-positive cells upon β-HCH stimulation. Fluorescence intensity was then quantified by averaging across the CTCF (Corrected Total Cell Fluorescence) calculated with ImageJ on the same number of cells for both control and treated samples from different images. The increase in fluorescence intensity after 48 h of treatment with β-HCH is visualized in the histogram. Images for both CTRL and β-HCH samples were captured under the same acquisition parameters and were background subtracted before analysis; the selected images are representative of three independent experiments. Results are presented as the mean and standard deviation. Statistical analysis was performed with GraphPad Prisma software using Student’s *t*-test. Statistically significant differences (*** *p* < 0.001) are marked with asterisks.

The transition of non-cancerous BEAS-2B cells into a malignant phenotype was further supported by analysis of the marker Ki67, a predictive indicator that clinically correlates with advanced tumor stages and poor prognosis. The expression of the nuclear protein Ki67 is strongly associated with proliferating cells and constitutes a routine investigation tool for the assessment of cancer aggressiveness [31]. Immunofluorescence staining revealed a noticeable increase in Ki67-positive cells after treating BEAS-2B with 10 µM β-HCH for 24 h. The fluorescence intensity was quantified for the same number of cells from different images for both control and treated samples; the variation in fluorescence intensity is displayed in the histogram in Figure 11.

### 2.9. β-HCH Induces Colony Formation

**Figure 12:**
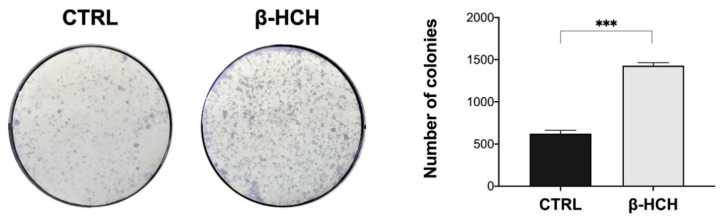
Impact of β-HCH on BEAS-2B cells’ clonogenic potential. Cells were seeded at a density of 200 cells/mL in a 6-well plate and treated for one week with 10 μM β-HCH. Then, cells were fixed with MeOH and stained with crystal violet, and the obtained colonies were quantified by using ImageJ software. As schematized in the figure, β-HCH induced an increase in the colony number, confirming its effects on cell proliferation. Experiments were repeated three times with similar results, and the obtained values are presented as the mean and standard deviation. Statistical analysis was performed with GraphPad Prisma software using Student’s *t*-test. Statistically significant differences (*** *p* < 0.001) are marked with asterisks.

The clonogenicity assay is a sensitive experimental approach employed to assess the proliferative characteristics of in vitro cells by measuring the capability of a single cell to form a colony [32]. In fact, the increase in colony number provides significant information about the differentiation status of a cell population. In order to confirm the influence of β-HCH on BEAS-2B cells’ clonogenic potential, cells were treated with 10 µM β-HCH for one week, and the number of obtained colonies was determined by staining them with crystal violet. The number of colonies in the sample exposed to β-HCH was more than doubled compared to that in the control (Figure 12); the outcome of the clonogenicity assay represents conclusive evidence of β-HCH’s effect on cells’ proliferative activity.

## 3. Discussion

Cancer is a condition intrinsically related to the physiologic and genetic features of mammals and was firstly identified in humans 4000 years ago [33]. Currently, there is growing scientific evidence that cancer is not only an endogenic process, but it may also be caused by exogenic factors [34]. The proper functioning of every single cell is required to ensure the well-being of the entire organism, and a perturbation in the delicate balance between living organism and their ecosystem could lead to the disruption of cellular homeostasis. Based on their influence on cancer development, environmental factors have been classified into [35]:

1. Primary determining factors, which comprise chemical substances, physical agents, and the outcomes of viral-induced carcinogenic transformation. All these three components especially act on signaling pathways and nucleic acids (DNA and RNA), hence the modern notion of cancer as a molecular disease;
2. Secondary determining factors, represented by hereditary determinism;
3. Favoring factors, which are risk factors whose occasional or systematic intervention has been observed in the incidence of malignant tumors. In this group, some geographic factors, nutrition factors, sex, age, etc., can be mentioned.

On the basis of this classification, environmental contaminants represent one of the main contributors to tumor initiation, as abundantly documented by the increasing number of research studies during the past decade [36]. The list of chemical agents catalogued as carcinogenic includes the different conformers of hexachlorocyclohexane, among which stands out the well-known pesticide Lindane (γ-HCH) [37]. The problems connected with the extensive use of Lindane for agricultural purposes have drawn the attention of the scientific community, but this is just the tip of the iceberg: the industrial synthesis of Lindane generated large quantities of undesired isomers that constitute a significant source of contamination [17]. The ecological disaster caused by large-scale Lindane production in the 1950s and 1960s is concretely evidenced by the considerable environmental residual amounts of β-hexachlorocyclohexane, the most bioaccumulative, stable, and persistent member of the HCH family [38]. Even though β-HCH is present worldwide in the form of massive physical stockpiles, little information is available on its biochemical and toxicological profile; this lack of knowledge reinforces the need to focus research efforts not only on developing remediation and degradation strategies, but also on considering the possible adverse impacts of β-HCH on human health.

Previous cellular studies from our laboratory investigated β-HCH’s activity on a panel of different cancer cell lines with the purpose of clarifying its biological effects. The obtained results demonstrated the capability of β-HCH to promote tumor progression through several molecular processes, which include STAT3 canonical and non-canonical activation, endocrine disruption, Aryl Hydrocarbon Receptor activation, and modulation of redox homeostasis and energy metabolism [19,20].

In this manuscript, we examined the potential role of β-HCH in tumor initiation by performing experiments on a human normal bronchial epithelium cell line, BEAS-2B. The experimental concentration of 10 µM for β-HCH is the same as that already employed in our previous works and corresponds to the average plasma levels detected in a population-based epidemiologic study in the Valle del Sacco [21]. It is well established in the literature that chemical substances may cause carcinogenesis via both genotoxic and non-genotoxic mechanisms by altering cellular signaling networks or inducing DNA damage [39].

Firstly, our study evidenced the proliferative activity of β-HCH: MTT assay revealed a 20–40% increase in cell viability, suggesting the activation of oncogenic transduction pathways related to the higher proliferation rate.

In support of this hypothesis, Western blot analysis carried out on total protein extracts from BEAS-2B cells exposed to 10 µM of β-HCH revealed the activation of the HER2-ERK1/2 axis already after 30 min. The HER2/ERK pathway can induce and sustain an autocrine signaling mechanism associated with the aberrant synthesis of growth factors, as confirmed by the presence of EGF in the culture media of BEAS-2B treated with β-HCH for 24–48 h. This β-HCH-dependent proliferative loop is accountable for the overproduction of ROS after 48 h which may, in turn, induce genotoxic responses via DNA damage.

For this reason, BEAS-2B cells were analyzed for γH2A.X by Western blot, and experimental outcomes evidenced an augmented phosphorylation of H2A.X from 12 to 72 h of incubation with 10 µM β-HCH. The occurrence of DNA damage was further confirmed by performing a comet assay that highlighted the increase in DNA fragmentation after 72 h of treatment. Both the phosphorylation of H2A.X and the DNA fragmentation are temporally coherent with indirect genotoxic activity of β-HCH and confirmed its effective role in the first step of carcinogenesis. In addition, β-HCH exhibited antiapoptotic properties and induced a slight accumulation of cells in the G2M phase, which is probably due to a delay in the cell cycle duration caused by the activation of the G2 checkpoint, responsible for the regulation of DNA repair mechanisms. The alteration of all these processes is reflected in the different growth modalities and cytoskeleton organization displayed by BEAS-2B stimulated with β-HCH, also testified to by the rearrangement of the structural protein vimentin. The ultimate proof to confirm the impact of β-HCH on cancer initiation is the enhanced proliferative activity attested to by the increased expression of Ki67, a prognostic marker associated with clinically advanced tumors, and the higher number of colonies obtained from the clonogenic assay.

## 4. Materials and Methods

### 4.1. Cell Culture

The human normal bronchial epithelium cell line BEAS-2B was obtained from the American Type Culture Collection (ATCC). Cells were grown to 80% confluence at 37 °C in 5% CO_2_ in the appropriate culture medium, RPMI 1640 (Sigma-Aldrich, cat. R0883) supplemented with 1% sodium pyruvate (Sigma Aldrich, Milano, Italy, cat. S8636), 10% fetal bovine serum (Sigma Aldrich, cat. F7524), 2 mM glutamine, 100 μg/mL streptomycin, and 100 U/mL penicillin (Sigma Aldrich, cat. P4333). Beta-hexachlorocyclohexane (β-HCH) (Sigma-Aldrich, 33376) was tested on each cell line at a final concentration of 10 μM.

### 4.2. Cell Viability

The impact of β-HCH on cell viability was evaluated by seeding 1.2 × 10^4^ cells/well in 96-well plates and determining cell viability after 24, 48, or 72 h of incubation with different concentrations of β-HCH (2, 10, 25, 50, and 100 μM). Cell viability was measured using MTT (3-(4,5-dimethylthiazol-2-yl)-2,5-diphenyl-2H-tetrazolium bromide) (Sigma-Aldrich, cat. M2128). Briefly, the culture medium was removed and 125 μL of MTT solution (0.5 mg/mL MTT in culture medium) was added to each well. After 3 h of incubation, the solution was removed, and the insoluble formazan dye, resulting from the conversion of tetrazolium salt by metabolically active cells, was dissolved by adding 125 μL/well of DMSO and measured at 570 nm using an Appliskan plate reader (Thermo Scientific, Monza, Italy).

### 4.3. Time Course Assay

BEAS-2B cells were seeded in a 6-well plate at a density of 3 × 10^5^ cells/well and treated with 10 μM β-HCH for from 30 min up to 24 h. Extracts were processed by immunoblotting and incubated with the indicated antibody. The stain-free blot image was used for total protein loading control and normalization.

### 4.4. Observation of Morphological Changes

BEAS-2B cells were seeded in a 6-well plate at a density of 2 × 10^5^ cells/well and treated with 10 μM β-HCH for 48 h; morphological changes were observed using an inverted microscope (Leica, Milano, Italy). In addition, cells were seeded on coverslips at a density of 2 × 10^2^ cells/mL and immunoassayed for the mesenchymal marker vimentin following the immunofluorescence procedure described below.

### 4.5. Protein Extraction and Immunoblotting

Protein extraction and immunoblotting analysis were performed essentially according to Rubini et al. [20]. Cells cultured on 6-well plates were scraped, harvested by centrifugation, and washed in PBS (Sigma Aldrich, cat. D8662). Total protein extracts were obtained using a lysis buffer containing 2% SDS (Bio-Rad, cat. 161030), 20 mM Tris-hydrochloride pH 7.4 (Sigma Aldrich, cat. T3253), 2 M urea (Sigma Aldrich, cat. U5378), 10% glycerol (Merck, Milano, Italy, cat. GE17-1325-01) added with 2 mM sodium orthovanadate (Sigma Aldrich, cat. S6508), 10 mM DTT (Sigma Aldrich, cat. D9779), and a protease inhibitor cocktail diluted 1:100 (Immunological Sciences, Roma, Italy, cat. IK-96010). Proteins were resolved by SDS-PAGE 10% TGX FastCastTM Acrylamide gel (BioRad, Segrate, Italy, cat. 161-0183) and transferred onto PVDF membranes using a Trans-Blot^®®^ TurboTM Transfer System (BioRad, cat. 170-4247). For the detection of histone H2AX, proteins were resolved by SDS-PAGE 7.5% Acrylamide gel (Sigma Aldrich, cat. A3699). The membranes were blocked with 3% BSA (Immunological Sciences, cat. ISP6154-100) or 0.2% *w*/*v* I-block (Thermo Fisher Scientific, T2015) in Tris-buffered saline containing 0.05% Tween-20 (Sigma Aldrich, cat. P7949) (TBS-T) and incubated with a specific primary antibody for 1 h. Subsequently, membranes were washed three times in TBS-T, then incubated for an additional hour with phosphatase-conjugated secondary antibody (Sigma-Aldrich, cat. A3687-A3688, dilution 1:5000). The alkaline phosphatase signal was detected with BCIP/NBT reagents (Carl Roth, Milano, Italy, cat. 6368.1 and 4421.3). The peroxidase signal was detected with ECL Fast Femto reagent (Immunological Science), acquired by a Molecular Imager^®®^ ChemiDoc™ MP System (Bio-Rad Laboratories), and the intensity of protein bands was quantified using ImageLab Software. Membrane images were acquired using the Molecular Imager^®®^ ChemiDoc™ MP System (Bio-Rad), and the intensity of protein bands was quantified using ImageLab Software. The immunoblotting detection was carried out using specific primary antibodies anti-pHER2 (Cell Signaling, Pero, Italy, cat. 2247S), anti-HER2 (Cell Signaling, cat. 2242S) anti-p^T202−204^p44/42 MAPK (p^T202−204^ERK1/2, Cell Signaling, cat. 9101S), anti-p44/42 MAPK (ERK ½, Cell Signaling, cat. 9102S), anti-pS^139^H2AX (Santa Cruz Biotechnology, Segrate, Italy, cat. 101696), and anti β-actin (Sigma Aldrich, cat. A1978 clone AC-15) diluted according to manufacturer’s instruction depending on the experiment.

### 4.6. Reactive Oxygen Species (ROS) Detection

Reactive oxygen species (ROS) generated by stressing cells with 10 μM β-HCH for 48 h or 75 μM tBHP for 1 h as a positive control were quantified using the CellROX Green Flow Cytometry Assay Kit (Thermo Fisher Scientific, cat. C10492) following the manufacturer’s instructions. Samples were analyzed using a BD Accuri C6 flow cytometer (BD Biosciences, Milano, Italy) and a fluorescent microscope (Leica AF6000 Modular System) with 20× objective.

### 4.7. Determination of Apoptosis

Cells were treated for 48 h with 10 μM β-HCH or 10 μM camptothecin (Sigma Aldrich, cat. 208925) as a positive control. Apoptosis was determined by flow cytometry using Annexin V-FITC (Immunological Sciences, cat. IK-11120) according to the manufacturer’s instructions. Samples were processed using a BD Accuri C6 flow cytometer (BD Biosciences) and results were analyzed using ModFit LT software.

### 4.8. Dot Blot

Pre-cut nitrocellulose membranes (Thermo Fisher Scientific, cat. 88018) and filter paper sheets were preliminary wetted in Tris-buffered saline (TBS); then, the dot blot apparatus (MilliBlot™ kit complete, Sigma Aldrich, cat. Z358800) was properly assembled and attached to a vacuum source with a waste trap. After rinsing the membrane with TBS, 500 μL of conditioned medium from BEAS-2B cells, treated for 24–48 h with 10 μM β-HCH, was spotted at the center of the grid, and vacuum was applied to load the sample onto the membrane. The membrane was blocked overnight in 0.2% *w*/*v* I-Block, and EGF was detected using a specific antibody against EGF (Invitrogen, Monza, Italy, cat. M805) following the colorimetric protocol described above.

### 4.9. Immunofluorescence

Immunofluorescence analysis was performed essentially according to Cocchiola et al. [40]. Cultured cells were grown on coverslips and treated with β-HCH for 48 h. Cells grown on coverslips were washed with PBS, fixed with 4% formaldehyde for 15 min, and then rinsed with PBS (Sigma Aldrich, cat. D8662). Cells were permeabilized with cold methanol (−20 °C) for 5 min. After washing three times with PBS, the cells were blocked overnight with 3% *w*/*v* BSA (Immunological Sciences, cat. ISP6154-100) in PBS. Fixed cells were processed by immunofluorescence staining using specific primary antibodies against vimentin (Cell Signaling, 5741S) and Ki-67 (Cell Signaling, cat. 9027S), properly diluted in PBS containing 2% *w*/*v* BSA for 1 h. Following three washes with PBS added with 0.05% Triton and 2% *w*/*v* BSA (PBS-T), cells were incubated for 1 h in darkness with an FITC-conjugated secondary antibody (Jackson Immunoresearch, Milano, Italy, AlexaFluor 488-conjugated, cat. 211-545-109, dilution 1:800). Cell nuclei were counterstained with 100 ng/mL Hoechst (Sigma Aldrich, cat. 94403) for 15 min. After washing with PBS-T, coverslips were mounted on glass microscope slides with DuolinkTM Mounting Medium and examined using a fluorescence microscope (Leica AF6000 Modular System) with 63× oil immersion objective. Samples were captured under the same acquisition parameters and were background subtracted before analysis. The fluorescence intensity was quantified by averaging across the CTCF (Corrected Total Cell Fluorescence) calculated via ImageJ on the same number of cells for both control and treated samples from different images according to McCloy et al. [41].

### 4.10. Cell Cycle Analysis

In order to analyze the cell cycle by flow cytometry, cells were treated with 10 μM β-HCH for 48 h. Then, after detaching them with trypsin, cells were washed with HBSS (Sigma Aldrich, cat. 55021C) and fixed with 70% cold ethanol (Fluka, cat.02860). Ethanol was added dropwise to the pellet while mixing by inversion. Samples were fixed to 30 min at 4 °C and then washed twice in HBSS. RNase (Sigma Aldrich, cat. R6513) was then added at a final concentration of 0.2 mg/mL and incubated for 5 min at 37 °C. Then, 60 μg/mL final concentrated propidium iodide (Sigma Aldrich, cat. P4170) was added and samples were incubated for 45 min at 37 °C in the dark. Before flow cytometry analysis, samples were centrifuged and resuspended in the proper volume of HBSS. Samples were analyzed using a BD Accuri C6 flow cytometer (BD Biosciences). The results were analyzed using ModFit LT software.

### 4.11. Comet Assay

BEAS-2B cells were seeded on a 6-well plate at a density of 4 × 10^4^ cells/well and were treated with 10 μM β-HCH for 24–72 h; cells stimulated with 75 μM tert-butyl hydroperoxide for 1 h were used as a positive control for DNA damage. Then, alkaline comet assay was performed essentially according to Lu et al. [42].

Briefly, slides were preliminarily coated with 1% agarose (Sigma Aldrich, cat. A0576) and were left to air-dry overnight. BEAS-2B cells were digested in trypsin for 3 min, and the trypsin was then neutralized using the proper volume of RPMI medium. After centrifugation, cells were resuspended at a ratio of 1:10 (*v*/*v*) in 1% low-melting-point agarose (Millipore, cat. 2070 OP), and 20 μL of the cells/agarose mixture was pipetted onto a slide and incubated overnight in the lysis solution (LS) (2.5 NaCl, 100 mM EDTA, 10 mM Tris-base, 200 mM NaOH, 1% sodium lauryl sarcosinate, and 1% Triton X-100) at 4 °C. Then, slides were immersed for 1 h at 4 °C in the dark in Alkaline Electrophoresis Solution pH > 13 (AES) (200 mM NaOH, 1 mM EDTA) to allow for DNA unwinding. Electrophoresis was carried out at 30 mA for 1 h at 4 °C. Then, slides were rinsed twice with dH2O and were immersed in 70% EtOH for 30 min at room temperature. After drying for 15 min at 37 °C in the dark, slides were stained with propidium iodide (Sigma Aldrich, cat. P4170) at a final concentration of 5 μM/mL (15 min at room temperature), rinsed with dH_2_O, and dried for acquisition. Images were captured using a fluorescence microscope (Leica AF6000 Modular System) with 20× objective and analyzed using CaspLab software.

### 4.12. Colony Formation Assay

BEAS-2B cells were seeded at a density of 2 × 10^2^ cells/mL in a 6-well plate and treated for one week with 10 μM of β-HCH. The medium was removed, then cells were rinsed with PBS and fixed with cold MeOH for 30 min at 4 °C. Thereafter, colonies were stained by incubating cells with a mixture of 1% crystal violet in 25% MeOH for 1 h at room temperature. After removal of the staining solution, each well was washed with abundant dH_2_O and air-dried at room temperature. Colonies were counted using ImageJ software according to Cai et al. [43].

### 4.13. Statistical Analysis

The repeatability of results was confirmed by performing all experiments at least three times. The obtained values are presented as the mean and standard deviation. Statistical analysis was performed with GraphPad Prisma software using Student’s *t*-test.

## 5. Conclusions

**Figure 13:**
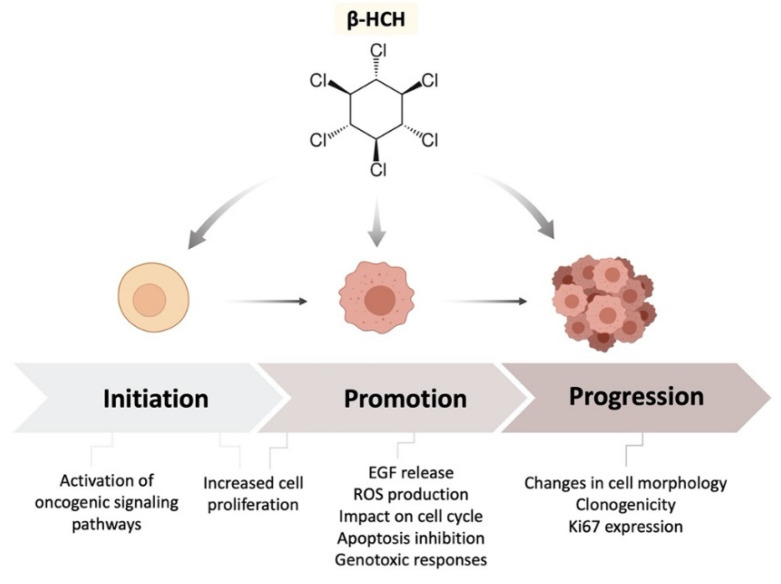
Schematic summary of β-HCH’s impact on all three stages of carcinogenesis.

The results from this study strongly support the carcinogenic potential of β-HCH, which is achieved through both non-genotoxic (proliferative and antiapoptotic activity) and indirect genotoxic (ROS production and DNA damage) mechanisms. Taking into account all these experimental data, together with the information provided by our previous studies carried out on different cancer cell lines, it can be concluded that β-HCH is able to act on all three stages of carcinogenesis: initiation, promotion, and progression (Figure 13). In light of its multifaceted biochemical effects and its intrusive presence in the environment, β-HCH should become the focus of multidisciplinary studies.

## Data Availability

Data is contained within the article.

## References

[1] Casey S.C., Vaccari M., Al-Mulla F., Al-Temaimi R., Amedei A., Barcellos-Hoff M.H., Brown D.G., Chapellier M., Christopher J., Curran C.S. (2015). The effect of environmental chemicals on the tumor microenvironment. Carcinogenesis.

[2] Fu X., Xu J., Zhang R., Yu J. (2020). The association between environmental endocrine disruptors and cardiovascular diseases: A systematic review and meta-analysis. Environ. Res..

[3] Thong T., Forté C.A., Hill E.M., Colacino J.A. (2019). Environmental exposures, stem cells, and cancer. Pharmacol. Ther..

[4] Sharma S., Wakode S., Sharma A., Nair N., Dhobi M., Wani M.A., Pottoo F.H. (2020). Effect of environmental toxicants on neuronal functions. Environ. Sci. Pollut. Res..

[5] Blackadar C.B. (2016). Historical review of the causes of cancer. World J. Clin. Oncol..

[6] Yamagiwa K., Ichikawa K. (1977). Experimental Study of the Pathogenesis of Carcinoma. CA Cancer J. Clin..

[7] Zheng W., McLaughlin J.K., Chow W.-H., Chien H.T.C., Blot W.J. (1993). Risk Factors for Cancers of the Nasal Cavity and Paranasal Sinuses among White Men in the United States. Am. J. Epidemiol..

[8] Ward E., Carpenter A., Markowitz S., Roberts D., Halperin W. (1991). Excess Number of Bladder Cancers in Workers Exposed to Ortho-Toluidine and Aniline. J. Natl. Cancer Inst..

[9] Ledda C., Rapisarda V. (2020). Occupational and Environmental Carcinogenesis. Cancers.

[10] Kahn L.G., Philippat C., Nakayama S.F., Slama R., Trasande L. (2020). Endocrine-disrupting chemicals: Implications for human health. Lancet Diabetes Endocrinol..

[11] Saravi S.S.S., Dehpour A.R. (2016). Potential role of organochlorine pesticides in the pathogenesis of neurodevelopmental, neurodegenerative, and neurobehavioral disorders: A review. Life Sci..

[12] Jayaraj R., Megha P., Sreedev P. (2016). Organochlorine pesticides, their toxic effects on living organisms and their fate in the environment. Interdiscip. Toxicol..

[13] Oliveira P.A., Colaço A., Chaves R., Guedes-Pinto H., De-La-Cruz P., Louis F., Lopes C. (2007). Chemical carcinogenesis. An. Acad. Bras. Cienc..

[14] Burgio E., Piscitelli P., Colao A. (2018). Environmental Carcinogenesis and Transgenerational Transmission of Carcinogenic Risk: From Genetics to Epigenetics. Int. J. Environ. Res. Public Health.

[15] Olivero-Verbel J., Guerrero-Castilla A., Ramos N.R. (2011). Biochemical Effects Induced by the Hexachlorocyclohexanes. Rev. Environ. Contam. Toxicol..

[16] Vijgen J., Abhilash P.C., Li Y.F., Lal R., Forter M., Torres J.P.M., Singh N., Yunus M., Tian C., Schäffer A. (2011). Hexachlorocyclohexane (HCH) as new Stockholm Convention POPs—A global perspective on the management of Lindane and its waste isomers. Environ. Sci. Pollut. Res..

[17] Vijgen J., de Borst B., Weber R., Stobiecki T., Forter M. (2019). HCH and lindane contaminated sites: European and global need for a permanent solution for a long-time neglected issue. Environ. Pollut..

[18] Li Y.-F., Macdonald R., Jantunen L., Harner T., Bidleman T., Strachan W. (2002). The transport of β-hexachlorocyclohexane to the western Arctic Ocean: A contrast to α-HCH. Sci. Total Environ..

[19] Rubini E., Altieri F., Chichiarelli S., Giamogante F., Carissimi S., Paglia G., Macone A., Eufemi M. (2018). STAT3, a Hub Protein of Cellular Signaling Pathways, Is Triggered by β-Hexaclorocyclohexane. Int. J. Mol. Sci..

[20] Rubini E., Paglia G., Cannella D., Macone A., Di Sotto A., Gullì M., Altieri F., Eufemi M. (2020). β-Hexachlorocyclohexane: A Small Molecule with a Big Impact on Human Cellular Biochemistry. Biomedicines.

[21] Narduzzi S., Porta D., Fantini F., Blasetti F., Davoli M., Forastiere F. (2016). Sorveglianza Sanitaria ed Epidemiologica della Popolazione Residente in Prossimità del Fiume Sacco, Rapporto Tecnico Attività 2013–2015.

[22] Malarkey D.E., Hoenerhoff M., Maronpot R.R. (2013). Carcinogenesis: Mechanisms and Manifestations. Haschek and Rousseaux’s Handbook of Toxicologic Pathology.

[23] Kakehashi A., Wei M., Fukushima S., Wanibuchi H. (2013). Oxidative Stress in the Carcinogenicity of Chemical Carcinogens. Cancers.

[24] Kumari S., Badana A.K., Murali Mohan G., Shailender G., Malla R.R. (2018). Reactive Oxygen Species: A Key Constituent in Cancer Survival. Biomark. Insights.

[25] Yang J., Zhao X., Tang M., Li L., Lei Y., Cheng P., Guo W., Zheng Y., Wang W., Luo N. (2017). The role of ROS and subsequent DNA-damage response in PUMA-induced apoptosis of ovarian cancer cells. Oncotarget.

[26] Nagelkerke A., Span P.N. (2016). Staining Against Phospho-H2AX (γ-H2AX) as a Marker for DNA Damage and Genomic Instability in Cancer Tissues and Cells. Chemistry and Biology of Pteridines and Folates.

[27] Speit G., Rothfuss A. (2012). The Comet Assay: A Sensitive Genotoxicity Test for the Detection of DNA Damage and Repair. Methods Mol. Biol..

[28] Alizadeh E., Castle J., Quirk A., Taylor C.D., Xu W., Prasad A. (2020). Cellular morphological features are predictive markers of cancer cell state. Comput. Biol. Med..

[29] Acheva A., Haghdoost S., Sollazzo A., Launonen V., Kämäräinen M. (2019). Presence of Stromal Cells Enhances Epithelial-to-Mesenchymal Transition (EMT) Induction in Lung Bronchial Epithelium after Protracted Exposure to Oxidative Stress of Gamma Radiation. Oxidative Med. Cell. Longev..

[30] Morrow C.S., Moore D.L. (2020). Vimentin’s side gig: Regulating cellular proteostasis in mammalian systems. Cytoskeleton.

[31] Mrouj K., Andrés-Sánchez N., Dubra G., Singh P., Sobecki M., Chahar D., Al Ghoul E., Aznar A.B., Prieto S., Pirot N. (2021). Ki-67 regulates global gene expression and promotes sequential stages of carcinogenesis. Proc. Natl. Acad. Sci. USA.

[32] Rafehi H., Orlowski C., Georgiadis G.T., Ververis K., El-Osta A., Karagiannis T.C. (2011). Clonogenic Assay: Adherent Cells. J. Vis. Exp..

[33] Faltas B. (2010). Cancer is an ancient disease: The case for better palaeoepidemiological and molecular studies. Nat. Rev. Cancer.

[34] Klaunig J. (2020). Chapter 8 – Carcinogenesis. An Introduction to Interdisciplinary Toxicology.

[35] Madia F., Worth A., Whelan M., Corvi R. (2019). Carcinogenicity assessment: Addressing the challenges of cancer and chemicals in the environment. Environ. Int..

[36] Venkatesh H.N., Manoj M.J., Ghosh D., Chetan G.K. (2015). Environmental pollutants leading to carcinogenesis: Process of natural selection of human cells due to chronic inflammation and sustained stress environment. Int. J. Environ. Sci. Technol..

[37] Krewski D., Bird M., Al-Zoughool M., Birkett N., Billard M., Milton B., Rice J.M., Grosse Y., Cogliano V.J., Hill M.A. (2019). Key characteristics of 86 agents known to cause cancer in humans. J. Toxicol. Environ. Health Part B.

[38] Caudle W.M. (2015). Occupational exposures and parkinsonism. Neurocutan. Syndr..

[39] Kripke M., Brody J.G., Hawk E., Hernandez A.B., Hoppin P.J., Jacobs M.M., Rudel R.A., Rebbeck T.R. (2020). Rethinking Environmental Carcinogenesis. Cancer Epidemiol. Biomark. Prev..

[40] Cocchiola R., Rubini E., Altieri F., Chichiarelli S., Paglia G., Romaniello D., Carissimi S., Giorgi A., Giamogante F., Macone A. (2019). STAT3 Post-Translational Modifications Drive Cellular Signaling Pathways in Prostate Cancer Cells. Int. J. Mol. Sci..

[41] McCloy R., Rogers S., Caldon C.E., Lorca T., Castro A., Burgess A. (2014). Partial inhibition of Cdk1 in G2phase overrides the SAC and decouples mitotic events. Cell Cycle.

[42] Lu Y., Liu Y., Yang C. (2017). Evaluating In Vitro DNA Damage Using Comet Assay. J. Vis. Exp..

[43] Cai Z., Chattopadhyay N., Liu W.J., Chan C., Pignol J.-P., Reilly R.M. (2011). Optimized digital counting colonies of clonogenic assays using ImageJ software and customized macros: Comparison with manual counting. Int. J. Radiat. Biol..

